# Inclusion Characteristics in 95CrMo Steels with Different Calcium and Sulfur Contents

**DOI:** 10.3390/ma13030619

**Published:** 2020-01-30

**Authors:** Xiang Li, Xiao Long, Linzhu Wang, Shouhao Tong, Xiutao Wang, Yin Zhang, Yutang Li

**Affiliations:** 1 College of Materials & Metallurgical Engineering, Guizhou Institute of Technology, Guiyang 550003, China; lixiang8656@163.com (X.L.); tongshouhao2017@163.com (S.T.); xiutao4090@163.com (X.W.); 2 School of Materials and Metallurgy, Guizhou University, Guiyang 550025, China; Gzu_YinZhang1109@163.com (Y.Z.); DomYutangLi@163.com (Y.L.)

**Keywords:** inclusion, size, sulfide, equilibrated transformation

## Abstract

In order to study the effect of Ca and sulfur contents on the characteristics of inclusions, industrial experiments using 95CrMo steel were conducted. SEM-EDS detections and stereological analysis were used to probe the characteristics of inclusions, including their compositions, morphologies, size, number density, and distribution. The results indicate that there were mainly three types of inclusions in 95CrMo steel billets with 6–18 ppm Ca and 30–100 ppm S: inclusions with single-phased morphology mainly composed of oxides; isolated MnS/CaS-only inclusions; inclusions with multi-phased morphology. The three-dimensional inclusion size distribution suggests that there were more Type-1 inclusions with a small size in low S containing steels. The average diameter of all types of inclusions increased with increasing Ca or S content in 95CrMo steel, indicating that the formation of MnS and CaS coarsened their size. The density distribution of inclusions indicates that the more inclusions there are, the more easily they aggregate and collide. Moreover, it is presumably concluded that the formation of sulfide in the outer layer of oxide inclusions weaken the attraction between oxide inclusions. The equilibrated transformation and formation of inclusions during the cooling process of 95CrMo steel was discussed based on thermodynamic calculation. The equilibrated transformation trajectory of inclusions in 95CrMo steel during the cooling process was Ca_2_SiO_4_ + MgO → Ca_3_MgSi_2_O_8_ → Spinel + CaS, which was corresponding to the detected results. The precipitation regular of sulfide was obtained. The formation mechanism for three types of inclusions was discussed.

## 1. Introduction

Inclusions are inevitable during the deoxidation process of molten steel, which affect the performance and service life of steel materials significantly. In order to control the characteristics of inclusions, suitable calcium addition is always applied to modify the hard Al_2_O_3_-based inclusions into liquid or partially liquid inclusions, which is advantageous not only for diminishing their impact effect on the continuous casting and the properties of steel, but also for improving the manufacturing process, such as reducing the nozzle clogging possibility. Some calcium addition is useful to modify the morphology of MnS, such as changing strip shaped MnS to spindle shaped, which promoting steel isotropy [1,2,3,4,5]. 

There are extensive researches on the formation mechanism and evolution of calcium aluminates [6,7,8,9,10,11]. Many studies were focused on the kinetic calculation of inclusions during calcium treatment [6,12]. Thermodynamic calculations also have been performed to predict the type of calcium aluminates and reliable stability phase diagram of various calcium aluminates were obtained [13]. However, the reactions in the molten steel are complex and inclusions containing CaS would form as the existence of S and CaS inclusions are undeformable [14,15]. Verma and Pistorius et al. [16,17] investigated the composition evolution of inclusions in liquid steel modified by calcium and found that CaS may form as an intermediate reaction product, which can subsequently react with Al_2_O_3_ to form calcium aluminates as reaction [1]. Zhang et al. [18] proposed that the content of sulfur content in steel affected the transient evolution of inclusions and there were more CaS–CaO formed just after the addition of calcium, and then the CaS content decreased in the case of sulfur more than 90 ppm. Yang et al. [19] studied the inclusion evolution after calcium addition in low carbon Al-killed steel with ultra low sulfur content and found that the CaS/CaO ratio of the inclusions increases linearly by increasing S/total oxygen (T.O.) of the steel. It can be concluded that the evolution of inclusions during calcium treatment are dependent on the content of sulfur and calcium in the steel. Moreover, oxide–sulfide duplex inclusions are always observed in the steel. Sridhar et al. [20] observed an inclusion core surrounded by a ring composed of Ca and S. Choudhary et al. [21] developed a thermodynamic model for predicting the formation of oxide–sulfide duplex inclusions arising out of competitive reactions between [O], [S], and [Ca] in Al-killed steel. Shin et al. [15] discussed the formation mechanism for “spinel + CaS” complex inclusions.

3 (CaO) + 2 [Al] + 3 [S] → 3CaS + Al_2_O_3_. (1)

The modification of inclusions by calcium treatment and formation of oxide–sulfide duplex inclusions affect the size of inclusions significantly, which has an effect on the fatigue life of steel [22]. Lots of researches have been focused on the effect of Ca content and on the size and number of calcium aluminate inclusions. Wang et al. [23] observed the low melting temperature CaO–Al_2_O_3_ inclusions of 10–20 μm in cast slabs with calcium treatment and found that it can be deformed into the stringer shaped B type inclusions longer than 150–350 mm. Hu et al. [24] proposed that with the appropriate calcium treatment, the number and size of inclusions in spring steel decreased from 5.8 per mm^−2^ and 3.9 μm to 4.8 per mm^−2^ and 2.5 μm, and the inclusions changed to have a small and dispersed distribution. Wu [25] concluded that the number of inclusions in submicron scale increased as Ca content increased from 2 ppm to 25 ppm in thick plates. However, limited studies were conducted about the effect of sulfur content on the size distribution of oxide–sulfide duplex inclusions. Oxide–sulfide duplex inclusions were also detected in the steel containing certain Mn, such as semi-free-cutting steels [26,27]. MnS precipitating during cooling and solidification can dissolve with CaS and generate a solid of (Mn, Ca) S [14]. The modification of inclusions by calcium treatment is more complex in this steel containing Al, Mn, and S. It is proposed Al_2_O_3_–CaS inclusions had a detrimental effect on the magnetic properties of a Ca-treated non-oriented electrical steel [28], but the complex inclusions mCaO.nAl_2_O_3_–(Mn, Ca) S with duplex phase can be a protective film on the rack face, which improves the durability of the tool [29].

95CrMo hollow steel is made for drill rods because of its high fatigue resistance, good toughness, strong anti-vibration performance, and low notch sensitivity. It is widely used in quarries, open pit mines, and construction sites [22,30]. With the application of a high-power rock drill, a higher requirement for the quality of the drill rod is needed. The hard Al_2_O_3_ inclusions are always detected in 95CrMo hollow steel and deteriorate the steel performance [31,32,33]. In order to decrease the impact of large and hard inclusions on the service life of the drill rod, the composition of inclusions in 95CrMo steel should be controlled. In this study, we aimed to modified the hard Al_2_O_3_ inclusions into complex inclusions mCaO.nAl_2_O_3_–(Mn, Ca)S, which reduced stress concentration between hard oxide inclusion with steel matrix and inhibit the initiation and propagation of cracks around hard oxide inclusion due to good plasticity of sulfide inclusion. However, the Ca content affects the formation of calcium aluminate inclusions and the morphologies of MnS. It is of great significance to control the sulfide and oxide inclusions in the steel and to improve the performance of the final steel product. Therefore, the effect of Ca and S on the characteristics of inclusions in the 95CrMo steel was studied in the current study. The transformation and form mechanism of different types of inclusions were discussed. The results will be helpful for improving the properties and fatigue life of drill rod steel and semi-free-cutting steels.

## 2. Materials and Methods

The industrial experiments were carried out in a certain steel plant in China. The steelmaking route for 95CrMo steel is “60t Consteel furnace → ladle refining (LF) → vacuum degassing (VD) refining → continuous casting”. Alumina was added into the ladle during the tapping process. Billets of 150 × 150 mm in size in four heats were collected in which different amounts of calcium wires and pyrite were added after VD refining at about 1540 °C to study the effect of calcium and sulfur contents on the characteristics of inclusions. The steel samples were denoted in Table 1 according to the addition of calcium wires and pyrite. The compositions of samples cut from billets were determined and are shown in Table 1.

The steel billets were machined into steel cuttings and cylinders (φ5 mm × 100 mm) for compositional analysis. Inductively Coupled Plasma-Atomic Emission Spectrometry method (ICP-AES) was used to analyze the contents of Al, Ca, and Mg. Total oxygen for three samples cut from each billet was detected by Leco TCH600 oxygen–nitrogen analyzer and the oxygen content in inclusions was estimated by Equation (2) [22]. Other compositions were obtained by ARL-4460 photoelectric direct reading spectrometry coming from the American Thermo Electron Corporation.

(2) $$\left[\%O{\left(M\right)}_{Insol}\right]=f_{v}\cdot \frac{\rho_{M_{x}O_{y}}}{\rho_{Fe}}\cdot \frac{yM_{o}\left(xM_{M}\right)}{M_{M_{x}O_{y}}}\times 10^{6},$$

where *f_V_* is the total volume fraction of oxide inclusions, *ρ_Fe_* is the density of Fe, and *ρ_MxOy_* is the density of the oxide inclusions (*ρ_Fe_ =* 7.8 g/cm^3^, *ρ_Al2O3_ =* 3.97 g/cm^−3^, *ρ_MgO_ =* 3.65 g/cm^−3^, *ρ_CaO_ =* 3.4 g/cm^−3^, *ρ_SiO2_ =* 2.65 g/cm^−3^, *ρ_Al2O3–MgO–CaO–SiO2_ = X_Al2O3_ ρ_Al2O3_ + X_MgO_ ρ_MgO_ + X_CaO_ ρ_CaO_ + X_SiO2_ ρ_SiO2_*) [34]. *M_MxOy_* and *X_MxOy_* are the molecular weight of *M_x_O_y_* and the molar fraction of *M_x_O_y_*.

The metallographic samples with 15 mm × 15 mm × 15 mm were cut from steel billets and were wet grinded to 2000 grits followed by diamond polishing for SEM-EDS detection of inclusions. Three metallographic samples were prepared for each billet and were all observed by light microscope roughly to choose one sample for each billet in which the distribution of inclusion is most inhomogeneous. Then the microphotographs were taken with SEM by choosing the scanning area where there are both inhomogeneous and homogeneous distributed inclusions. The continuous 50 SEM microphotographs were taken at a magnification of 1000 corresponding to the total area of 1.4 × 2.7 mm^2^ to analyze the planer characteristics of inclusions, including their size, number density, and central coordinates by the Image-ProPlus 6.0 software (Media Cybernetics, Rockville, MD, USA). The size of inclusions *D* is the equivalent diameter calculated by Equation (3) and A is the area of inclusion.

(3) $$D=2\sqrt{\frac{A}{\pi }}.$$

The two-dimensional inclusion size distribution was transformed into three-dimensional inclusion size distribution based on stereological analysis (modified Schwartz–Saltykov method with the probability mass function [35,36]), which was applied in the estimation of the volume fraction inclusions in Equation (2), and the details are similar to our previous research [11,37]. The contents of Al, Ca, Mg, Si, Mn, O, and S in inclusions were detected by point EDS analysis in the center of each inclusion and they were transformed into their respective compound based on their stoichiometric relationship.

## 3. Results and Discussions

### 3.1. Morphologies and Compositions of Inclusion

**Figure 1:**
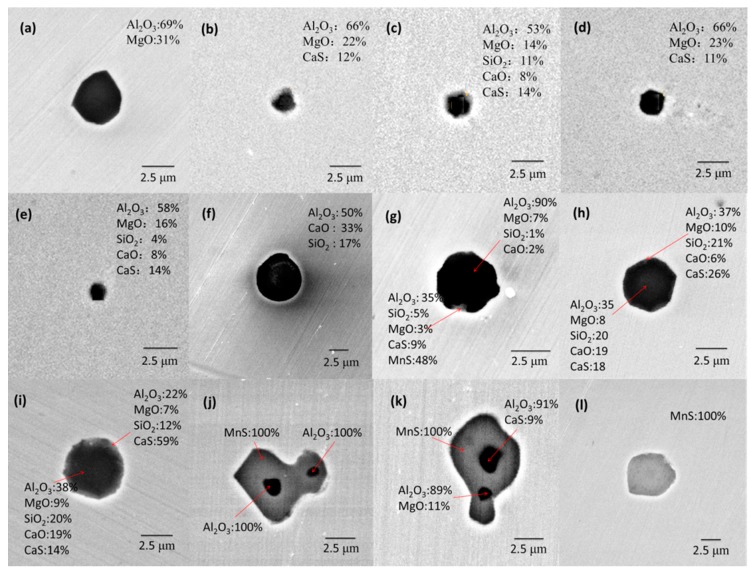
Morphologies and compositions of typical inclusions in samples. (**a**–**d**) Type-1 inclusions; (**e**–**h**) Type-2 inclusions; (**i**–**l**) Type-3 inclusions.

**Figure 2:**
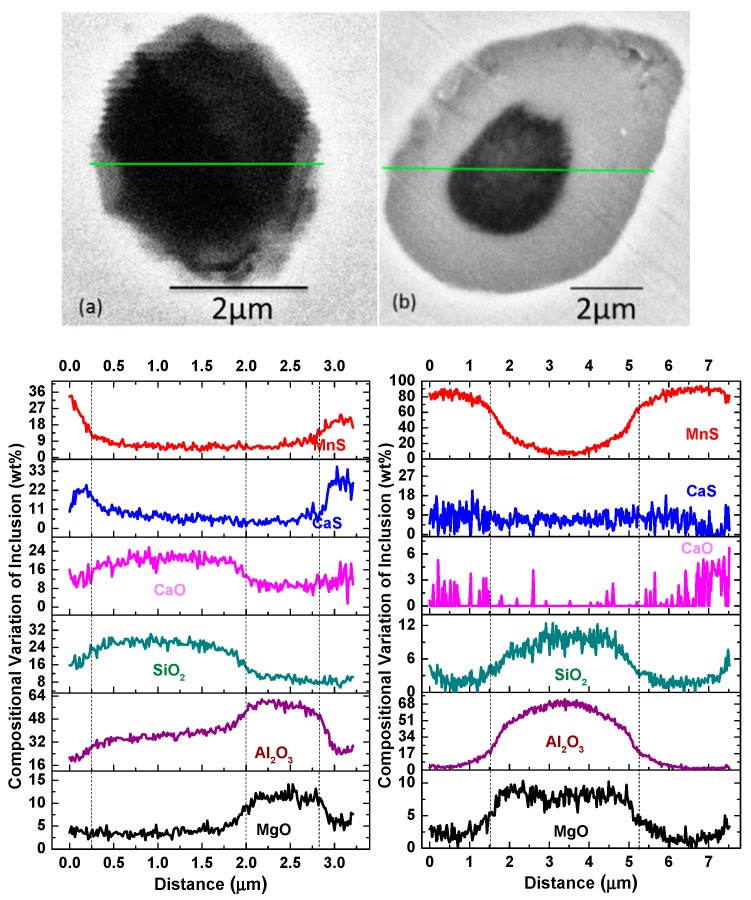
Line scanning of compositions for inclusions with multi-phased morphology. (**a**) SEM graph; (**b**) line scanning results.

Typical inclusions in all the samples observed by SEM-EDS are shown in Figure 1 and they can be classified into three types. Numerous inclusions with single-phased morphology were mainly composed of oxides with CaS less than 30%, such as spinel, Al_2_O_3_–CaO–SiO_2_ and Al_2_O_3_–MgO–CaO–SiO_2_–CaS (denoted Type-1 inclusion). Cr oxide in inclusions is no more than 5%, and thus it is not discussed in this study. Isolated MnS/CaS-only inclusions were also detected (denoted Type-2 inclusion) and the isolated CaS-only inclusions were only observed in Experiment 2. There were two kinds of inclusions with multi-phased morphology (denoted Type-3 inclusion), one being crystallizing complex CaS(–Mn)S adhering on oxides composed of Al_2_O_3_, MgO, CaO, and SiO_2_, another being MnS on oxides, which were mainly Al_2_O_3_, spinel, or complex oxides. Line scanning results of inclusions with multi-phased structure are shown in Figure 2, indicating a concentration gradient. The outer layer of inclusions was always rich in CaS and MnS. The inner layer was mainly composed of spinel, CaO–SiO_2_, or Al_2_O_3_–MgO–SiO_2_. Furthermore, it is found that the outer layer composed of MnS was thicker than that composed of CaS–MnS. The inclusions with multi-phased morphology and isolated MnS-only inclusions had a larger size compared with Type-1 inclusions.

**Figure 3:**
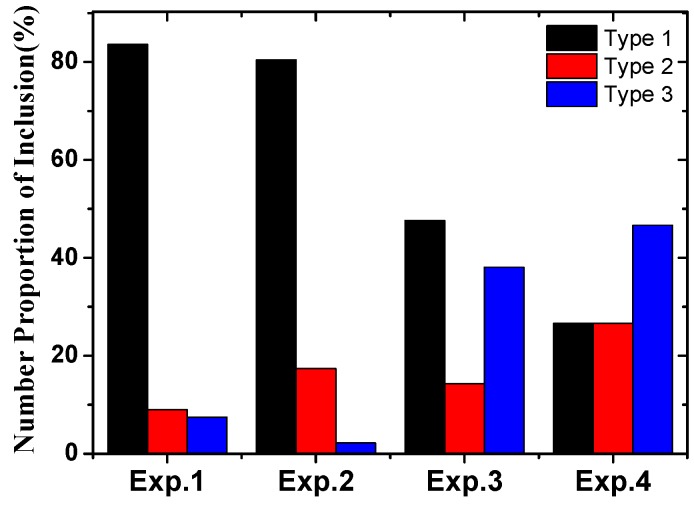
Proportion of three types of inclusions in each experiment.

The proportion of typical three kinds of inclusions in different experiments is illustrated in Figure 3. It is obvious that the experiments with low sulfur were rich in Type-1 inclusions and those with high sulfur were rich in Type-3 inclusions. The proportion of single-phased inclusions (Type-1 inclusion) was higher in the high Ca-containing experiments.

**Figure 4:**
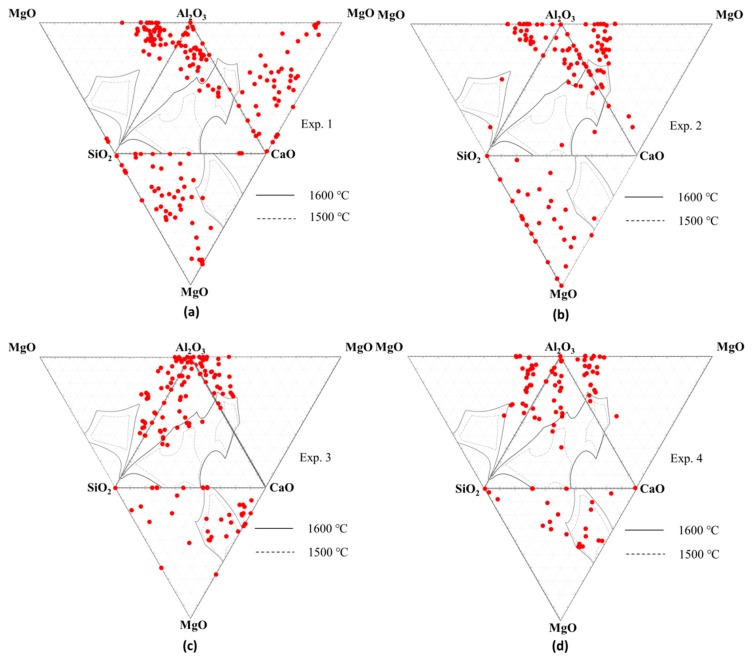
Composition distribution of inclusions in steel billets at different temperatures. (**a**) Experiment 1; (**b**) Experiment 2; (**c**) Experiment 3; (**d**) Experiment 4.

The composition distribution of oxides in Type-1 inclusions is displayed in Figure 4. It can be seen that the oxides were rich in Al_2_O_3_ and there were not many liquid oxides during the continuous casting process. The average CaO content in inclusions for high Ca-containing experiments (Experiments 1 and 3) was higher than that for low Ca-containing experiments (Experiments 2 and 4). The average MgO content in inclusions for Experiment 1 was higher than Experiment 2, and that for Experiment 3 was lower than Experiment 2. This is probably due to the floatation of inclusion into slag affecting the composition and properties of slag, and thus the slag with good liquidity erodes the refractory more seriously.

### 3.2. Size and Number Density of Inclusions

**Figure 5:**
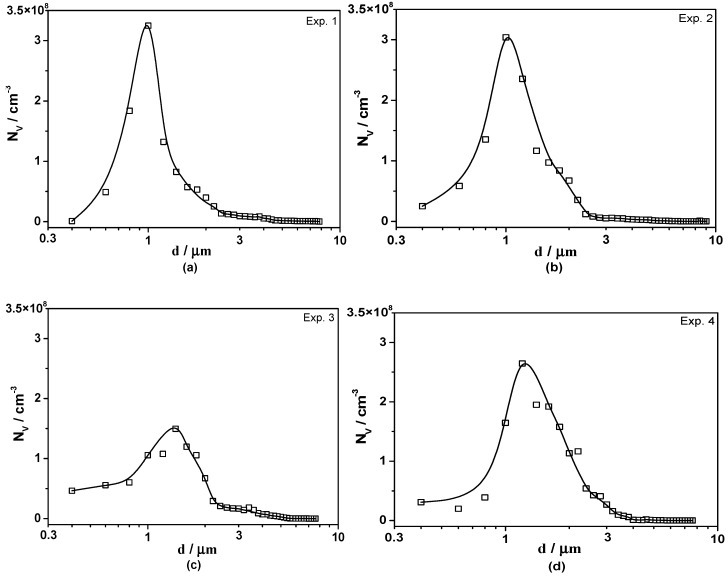
Three-dimensional inclusion size distribution with single-phased morphology. (**a**) Experiment 1; (**b**) Experiment 2; (**c**) Experiment 3; (**d**) Experiment 4.

The three-dimensional size distribution of Type-1 inclusions in different experiments was obtained based on stereological analysis, as shown in Figure 5. The inclusion size distribution seemed present in normal distribution and the size of inclusions ranged from about 0.4 to 9 μm. It is obvious that the peak points of curves corresponding to the diameter of inclusions in Experiments 1–4 are 1 μm, 1 μm, 1.4 μm, and 1.2 μm, respectively, and the height of curves in Experiments 3 and 4 is lower than that in Experiments 1 and 2, which indicates that there were more small Type-1 inclusions in low S containing steels.

**Figure 6:**
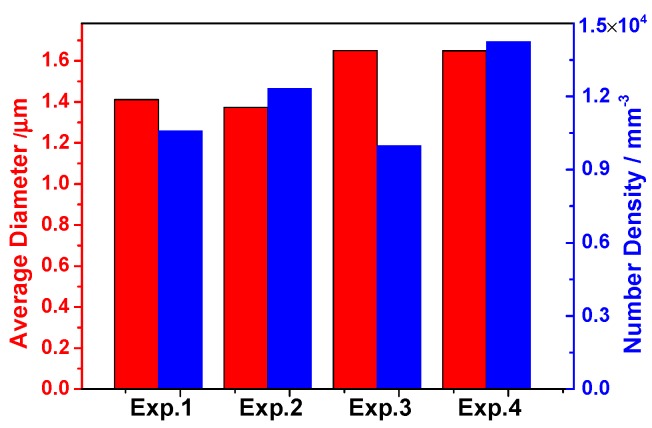
Three-dimensional average diameter and number density of Type-1 inclusions.

**Figure 7:**
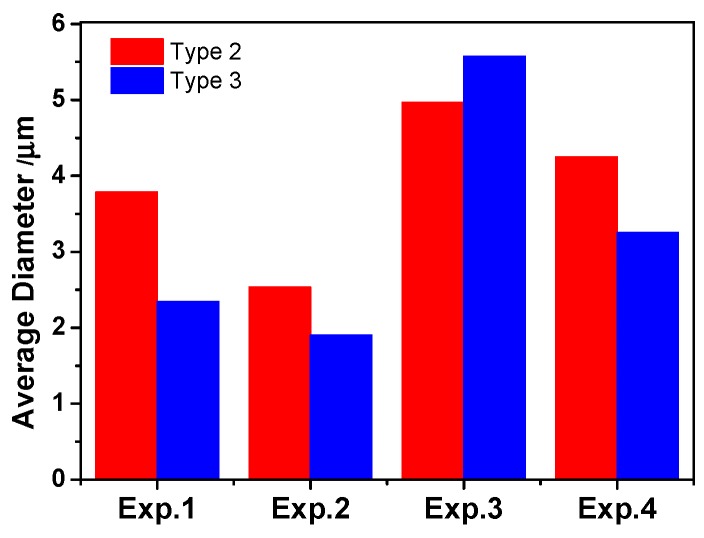
Three-dimensional average diameter of Type-2 and 3 inclusion.

Figure 6 suggests that the average diameter of Type-1 inclusions was larger in the steel with high S content probably attributed to the precipitation of sulfide in oxide inclusion, especially CaS. In addition, it is found that the number density of inclusions was larger in low Ca containing steel (Experiments 2 and 4). The three-dimensional average diameter of Type-2 and 3 inclusions is shown in Figure 7. Type-2 and 3 inclusions have a larger size than Type-1 inclusions. The average diameter of Type-2 inclusions had a same change trend with that of Type-3 inclusions, i.e., it was larger in the steel with high S or Ca content. It indicates that the formation of MnS and CaS also coarsens the size of Type-2 and 3 inclusions, and thus the inclusion size increased with increasing Ca or S content in 95CrMo steel on the whole.

### 3.3. Inclusion Distribution

**Figure 8:**
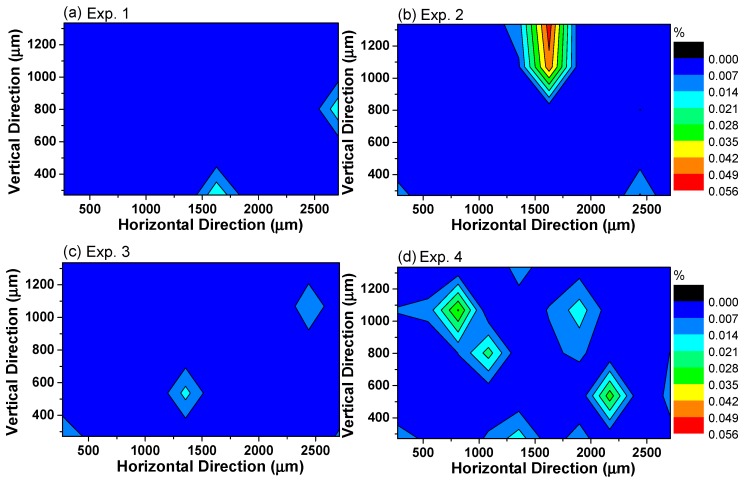
Density distribution of inclusions on the cross section in each experiment.

In order to study the distribution of inclusions in 95CrMo steel, the area of inclusions and their coordinates were measured by Image software combining with SEM detection. The area density (*A_d_*) distribution of inclusions on the cross section in each experiment is shown in Figure 8. The *A_d_* value is equal to $\frac{A_{inclusion}}{A_{steel\;sample}}\times 100\%$, i.e., the ratio of inclusion area and steel sample area. The inclusions seriously segregated in certain parts of the steel for Experiment 2 in which the largest area density of inclusions accounted for 0.049 to 0.056 pct. The inclusions were prone to segregate in Experiments 2 and 4 in which the number density of inclusions is large, indicating that the more inclusions there are, the more easily they aggregate and collide. Moreover, it is presumably concluded that the formation of sulfide in the outer layer of oxide inclusions weakens the attraction between oxide inclusions, and thus the inclusions in molten steel or semi-solidification steel with less sulfide tend to attract each other and aggregate strongly.

### 3.4. Formation Mechanism of Inclusions

**Figure 9:**
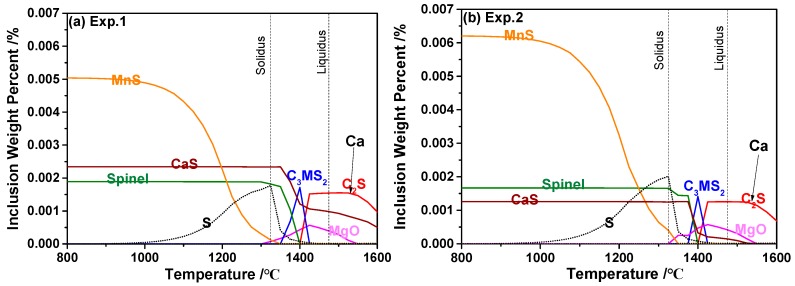

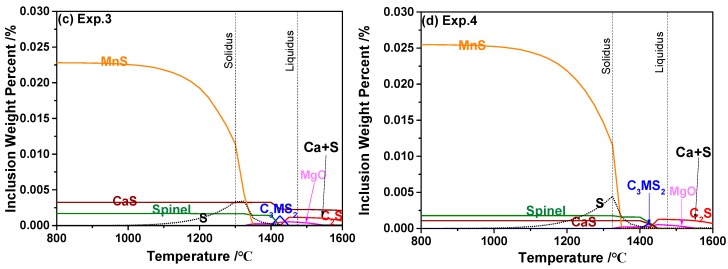
Phase transformation of inclusions and sulfur content in γ-Fe during cooling of 95CrMo steels by FACTSAGE (C_2_S represents 2CaO·SiO_2_; C_3_MS_2_ represents 3CaO·MgO·2SiO_2_). (**a**) Experiment 1; (**b**) Experiment 2; (**c**) Experiment 3; (**d**) Experiment 4.

The equilibrium precipitation of inclusions during cooling of 95CrMo was estimated by Factsage 7.2 with FToxid, FactPS, and FSstel data bases based on the steel compositions in Table 1, as displayed in Figure 9, and the adding temperature of Ca and S is marked in Figure 9. The same calculated method can be found in many studies [28,38,39]. However, the calculation by Factsage 7.2 is based on a normal equilibrium cooling model, and thus there may be a deviation of the calculation for temperature below solidus. It can be seen that the inclusions composed of Ca_2_SiO_4_, MgO, and CaS precipitated firstly in molten steel, which were transformed into Ca_3_MgSi_2_O_8_ during the solidification process of steel, and subsequently the inclusions composed of spinel and CaS formed and their amount increased due to the reaction [4]. Therefore, it can be concluded that the equilibrated transformation trajectory of inclusions in 95CrMo steel during the cooling process was Ca_2_SiO_4_ + MgO → Ca_3_MgSi_2_O_8_ → Spinel + CaS, which was corresponding to the detected Type-1 inclusions. The amount of spinel increased in the order of Experiment 2 = Experiment 3 < Experiment 4 < Experiment 1, which was in good agreement with the detected MgO content in oxides inclusions in Figure 4. Furthermore, the spinel and CaS formed during a solidification process tend to be small because the inclusions are hard to aggregate and collide in the melts with high viscosity. This meets the experimental results in Figure 1a–d where the inclusions composed of spinel and CaS had a small size of no more than 1.5 μm. The calculated results indicate that there were no inclusions containing Al_2_O_3_ except for spinel formed during the cooling process of 95CrMo, but the pure Al_2_O_3_ was observed in the billets experimentally. This is due to pure Al_2_O_3_ forming during the refining process and not having been modified.

(Ca_3_MgSi_2_O_8_) + 2[Al] + 3[S] = (Al_2_O_3_·MgO) + 3(CaS) + 2[Si] + 4[O]. (4)

The sulfide inclusions formed in the order of CaS > MnS. CaS formed in the molten steel or two-phase region of steel which adhered to the oxides (this type of inclusion can be seen in Figure 1h,i and Figure 2e), resulting in the coarsening of inclusions. The precipitation temperature of the CaS phase was higher in the steel containing high Ca and its amount increased with the increasing concentration of Ca and S. The thermodynamic calculated results suggest that the steel started to solidify from about 147 °C at which γ-Fe formed and S element segregated in austenite phase gradually during this process. The content of S in austenite phase increased sharply from about 1350 °C which promoted the formation of MnS inclusions. The amount of MnS inclusions kept increasing with the decreasing temperature. However, the concentration of S decreased with the increasing amount of precipitated MnS after the temperature of steel was lower than solidus temperature. In addition, it is found that the segregation of S in γ-Fe only promote the formation of MnS not CaS. The reason why the outer layer of multi-phased inclusions containing MnS was thicker than those containing CaS can be attributed to the fact that the amount of precipitated MnS was much larger than of CaS. Furthermore, the amount of MnS was obviously larger in the experiments with higher S concentration, which resulted in a higher proportion of inclusion with multi-phases in Experiments 3 and 4. The proportion of Type-3 inclusions was higher in high Ca-containing steel attributed to a larger amount of precipitated CaS phase.

**Figure 10:**
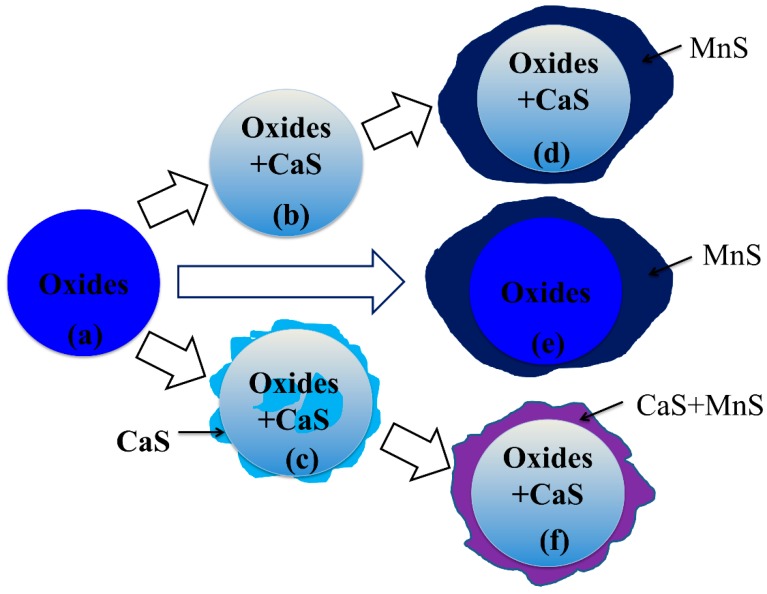
Schematic diagram for formation mechanism of Type-1 and 3 inclusions.

The formation mechanism of inclusions is illustrated in Figure 10 and different types of inclusions can be explained as following:

(1) Type-1 inclusions include pure oxides, as illustrated in Figure 10a, and the complex oxide–sulfide inclusions, as illustrated in Figure 10b. The pure oxides were formed during the refining process or precipitated during the cooling process. The inclusions in Figure 10b composed of oxides and CaS formed during the transformation process of inclusions as reaction [4]. The generated CaS dissolved in the oxides, and the solubility degree of CaS in calcium aluminate was high;
(2) Type-2 inclusions are pure MnS which nucleated homogeneously from about 1350 °C;
(3) Type-3 inclusions are inclusions with multi-phase, as illustrated in Figure 10c–f. The inclusions surrounded with a CaS shell always form when the amount of CaS increases to the extent that the CaS content is more than its supersaturation degree in oxide, as illustrated in Figure 10c. Some CaS detected in the core of inclusions may be due to the aggregation or uneven distribution of S during its dissolution in liquid steel. MnS nucleated around pure oxides and complex oxides–CaS inclusions heterogeneously after about 1350 °C, as illustrated in Figure 10d–f. The calculation of lattice disregistry based on Equation (5) [40] indicated that the disregistry between MgAl_2_O_4_ and MnS had the lowest value of 4.1 [15] and that for Al_2_O_3_ was 26.9, which facilitated the heterogeneous nucleation of MnS.
  (5) $$\delta_{{\left(hkl\right)}_{n}}^{{\left(hkl\right)}_{s}}=\sum_{i=1}^{3}\frac{1}{3}\cdot \frac{\left|\left(d_{{\left[uvw\right]}_{s}^{i}}\cdot \cos\theta -d_{{\left[uvw\right]}_{n}^{i}}\right)\right|}{d_{{\left[uvw\right]}_{n}^{i}}}\times 100,$$
  where (*hkl*)*_s_* is a low-index plane of the substrate, [*uvw*]*_s_* is a low-index direction in (*hkl*)*_s_* plane, (*hkl*)*_n_* is a low-index plane of the nucleated solid, [*uvw*]*_n_* is a low-index direction in (*hkl*)*_n_* plane, d[*uvw*]*_s_* is the distance between a nonmetallic element along [*uvw*]*_s_*, d[*hkl*]*_s_* is the distance between a nonmetallic element along [*hkl*]*_n_*, and *θ* is the angle between [*uvw*]*_s_* and [*uvw*]*_n_*.

## 4. Conclusions

Industrial experiments using 95CrMo were conducted to study the effect of calcium and sulfur contents on the characteristics of typical inclusions in steel, including their compositions, morphologies, size, number density, and distribution. The results are as follows:

(1) The three-dimensional inclusion size distribution suggests that there were more Type-1 inclusions with a small size in low S containing steels. The average diameter of all types of inclusions increased with increasing Ca or S content in 95CrMo steel, indicating that the formation of MnS and CaS coarsened their size;
(2) The density distribution of inclusions indicates that the more inclusions there are, the more easily they aggregate and collide. Moreover, it is presumably concluded that the formation of sulfide in the outer layer of oxide inclusions weaken the attraction between oxide inclusions;
(3) The thermodynamic calculation indicates that the equilibrated transformation trajectory of inclusions in 95CrMo steel during the cooling process is Ca_2_SiO_4_ + MgO → Ca_3_MgSi_2_O_8_ → Spinel + CaS. The precipitation temperature of the CaS phase was higher in the steel containing high Ca, and its amount increased with the increasing concentration of Ca and S. The segregation of S in γ-Fe promotes the formation of MnS, not CaS. Furthermore, the amount of MnS was larger in the experiments with higher S concentration;
(4) The formation mechanism on three types of inclusions was discussed. The inclusions surrounded with a CaS shell always formed when the amount of CaS increased to the extent that CaS content was more than its supersaturation degree in oxide. The low disregistry for MnS–spinel and MnS–Al_2_O_3_ facilitated the heterogeneous nucleation of MnS.

## Figures and Tables

**Table 1:** Chemical compositions of steel billets for 95CrMo.

Exp.	Remarks	C	Si	S	P	Mn	Mo	Cr	Alt	Als	Ca	Mg	O.T.	O_Insol_
			Weight Percent (%)						Mass (ppm)					
1	High Ca Low S	0.93	0.35	0.003	0.012	0.33	0.2	1.06	30	24	13	<5	8–9	7.6
2	Low Ca Low S	0.97	0.28	0.003	0.010	0.32	0.2	1.04	30	26	7	<5	6–9	7.3
3	High Ca High S	0.98	0.26	0.010	0.010	0.31	0.2	1.04	30	26	18	<5	7–8	7.7
4	Low Ca High S	0.94	0.23	0.010	0.013	0.31	0.2	1.03	30	19	6	<5	7–9	7.8

O.T. represents total oxygen and O_Insol_ represents oxygen in inclusions. Alt and Als represent the total alumina and soluble Al in the liquid steel, respectively.
